# Acute Pancreatitis: An Unusual Extrapulmonary Manifestation of Mycoplasma pneumoniae

**DOI:** 10.7759/cureus.25052

**Published:** 2022-05-16

**Authors:** Hina Rmsha Alfia Khan, Avtar Singh, Omer Usman, Samir Rafiq, Anam Amin

**Affiliations:** 1 Internal Medicine, Deccan College of Medical Sciences and Research Centre, Hyderabad, IND; 2 Internal Medicine, Swami Dayanand Hospital, New Delhi, IND; 3 Internal Medicine, Services Institute of Medical Sciences (SIMS), Lahore, PAK; 4 Anesthesia and Critical Care, Kabir Medical College, Peshawar, PAK; 5 Internal Medicine, Northwest General Hospital, Peshawar, PAK

**Keywords:** extrapulmonary symptoms, acute pancreatitis, mycoplasma pneumonia, m. pneumoniae, mycoplasma pneumoniae

## Abstract

*Mycoplasma pneumoniae* is a respiratory pathogen responsible for community-acquired atypical pneumonia. Apart from respiratory manifestations, other system involvement has also been reported. We present a case of interstitial pneumonia and a concurrent episode of acute pancreatitis in a young female who presented with fever, cough, vomiting, and epigastric pain. The abdominal evaluation revealed epigastric tenderness with no signs of organomegaly. Her complete metabolic profile was nonsignificant except for elevated serum lipase and amylase. Clinical, serological, and radiological features and detailed investigations confirmed the diagnosis of acute pancreatitis and interstitial pneumonia caused by *M*. *pneumoniae* in the absence of any other etiology. Her respiratory and gastrointestinal symptoms improved rapidly after commencing clarithromycin, providing a possible link between *M*. *pneumoniae* and pancreatitis.

## Introduction

*Mycoplasma pneumoniae* is a respiratory pathogen responsible for community-acquired atypical pneumonia that constitutes 20% of total community-acquired pneumonia [1]. Apart from respiratory manifestations, extrapulmonary involvement has also been reported. *M. pneumoniae* can affect the skin, gastrointestinal system, joints, central nervous system, and blood cells. *M. pneumoniae* can manifest as encephalitis, meningitis, autoimmune hemolytic anemia, urticaria, erythema nodosum, or arthralgia [2]. Gastrointestinal manifestations of *M. pneumoniae* include anorexia, vomiting, diarrhea, and abdominal pain and account for 25% of extrapulmonary manifestations caused by *M. pneumoniae* [3]. *M. pneumoniae* as an etiology of acute pancreatitis is rarely described in the literature [4-7]. Herein, we describe a case of acute pancreatitis and concomitant interstitial pneumonia caused by *M. pneumoniae*.

## Case presentation

A 41-year-old female with no significant past medical history was brought to the emergency department with epigastric pain, nausea, and vomiting for the last five hours. The pain was sharp, sudden in onset, 8/10 in intensity, radiated to the back, and accompanied by nausea and two episodes of non-bloody vomiting. She also complained of cough, fever, and mild dyspnea for the last three days, for which she took paracetamol. However, her symptoms had not resolved. She denied smoking, alcohol use, and illicit drug abuse. She reported no history of travel, sick contacts, or recent trauma.

On initial evaluation, she looked dehydrated and well oriented in time, place, and person. She had a temperature of 100^o^F, respiratory rate of 21/minute, blood pressure of 110/75 mmHg, and heart rate of 95/minute. The abdominal evaluation revealed epigastric tenderness with no signs of organomegaly. Respiratory and cardiovascular examination was unremarkable. The results of initial laboratory results are shown in Table 1. The complete metabolic profile was nonsignificant except for elevated serum lipase and amylase. The results of the lipid profile were within the normal range.

**Table 1:** Result of initial blood investigations. ESR: Erythrocyte sedimentation rate, ALP: Alkaline phosphatase, RBC: Red blood cell count, WBC: White blood cell count, CRP: C-reactive protein, ALT: Alanine aminotransferase, AST, Aspartate aminotransferase.

Parameter	Lab result
RBC	4.7 million cells/mm^3^ (4.45-5.65)
WBC	13900 cells/mm^3^ (4000-11000)
Platelet count	261000 cells/mm^3^ (150,000-350,000)
Hemoglobin	13 mg/dL (12-15.5)
Hematocrit	44% (35.5-44.9)
Serum amylase	553 IU/L (30-110)
Serum lipase	1821 IU/L (0-160)
ALT	32 IU/L (7-55)
AST	35 IU/L (8-48)
ALP	59 mg/dL (36-92)
Total bilirubin	1.8 mg/dL (0.3-1.2)
Serum creatinine	0.9 mg/dL (0.7-1.2)
Blood urea nitrogen	17 mg/dL (14-20)
Prothrombin time	11.5 second (11-13.5)
Partial thromboplastin time	32 seconds (30-40)
Serum calcium	9.8 mg/dL (9.0-10.5)
ESR	31 (0-22)
CRP	68 mg/L (0-5)
Serum sodium	137 mmol/L (135-145)
Serum potassium	4.0 mmol/L (3.6-5.2)

**Figure 1:**
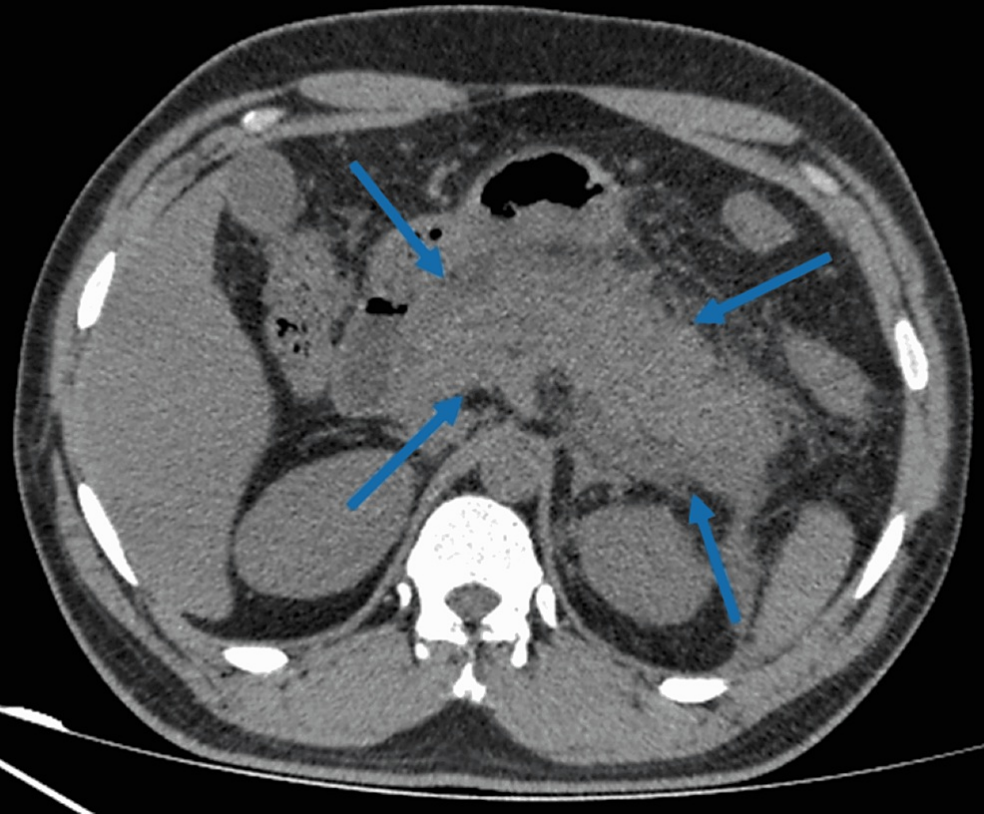
CT abdomen demonstrating diffuse inflammation of the pancreas with ill-defined borders (blue arrows).

Abdominal ultrasound was negative for any biliary ductal dilation, gall stones, or gall bladder inflammation. She underwent abdominal computed tomography (CT), which revealed swollen and edematous pancreas with ill-defined borders, consistent with a diagnosis of acute pancreatitis (Figure 1). The patient was kept nothing by mouth, and she was managed supportively with intravenous fluids, appropriate analgesics, and antiemetics.

**Figure 2:**
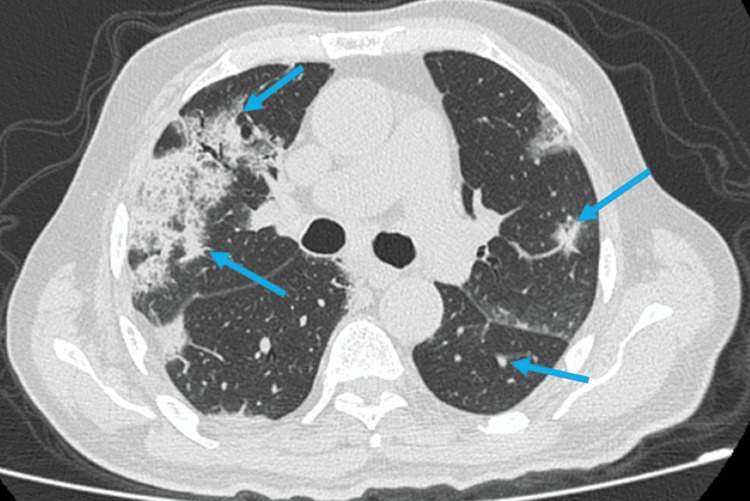
CT chest showing isolated infiltrates and consolidation in both lungs.

The following day, she reported worsening respiratory symptoms with inspiratory crackles on chest auscultation. The chest x-ray showed patchy interstitial infiltrates, and a high-resolution chest CT revealed scattered infiltrates and consolidation in both lungs (Figure 2). Blood cultures and polymerase chain reaction (PCR) were negative for influenza, parainfluenza, and coronavirus. Infectious workup and serology assays for human immunodeficiency virus, hepatitis A, B, C, and *Chlamydia psittaci* were unremarkable. Serological assay for *M. pneumoniae* revealed an elevated titer of immunoglobulin M (IgM) (33 UA/ml), consistent with recent infection, and serum titer was positive for cold agglutinin.

She was diagnosed with acute pancreatitis and diffuse interstitial pneumonia (DIP) caused by *M. pneumoniae*. She was commenced on clarithromycin 500 mg twice a day along with the supportive treatment. She was observed closely, and a daily assessment of her amylase and lipase levels was done. Her respiratory and abdominal symptoms improved over the next three to four days with complete resolution of symptoms and normalization of serum lipase and amylase levels.

## Discussion

Acute pancreatitis, an inflammatory disorder of the pancreas, is a leading cause of hospitalization for gastrointestinal disorders in many countries [8,9]. Acute pancreatitis is a potentially fatal disease with a mortality rate of 5% in the USA. Most patients with acute pancreatitis experience epigastric pain radiating to the back, which is frequently unbearable [8]. The pain is often associated with nausea and vomiting. It has been estimated that five to 80 cases of acute pancreatitis per 100,000 population are reported each year in the United States, and the incidence is rising due to alcohol abuse, gallstones, and obesity [9]. Infections, surgery, metabolic disorders, autoimmune disorders, and medication side effects can also trigger acute pancreatitis; however, 20% of the cases remain idiopathic [10]. Though infectious causes are quite rare, they include the herpes simplex virus, measles virus, varicella-zoster virus, mumps virus, *Salmonella*, *Mycoplasma*, and *Legionella* [11]. Acute pancreatitis caused by *M. pneumoniae* is not widely reported in the literature, and we have summarized cases of acute pancreatitis caused by* M. pneumoniae* in Table 1 [3-7]. Pancreatitis induced by *M. pneumoniae* can range from asymptomatic pancreatitis to acute necrotizing pancreatitis.

**Table 2:** Reported cases of acute pancreatitis caused by M. pneumoniae. CT: computed tomography, MP: M. pneumoniae, NR: not reported, M: male, F: female, IgM: Immunoglobulin M.

Author	Age/Sex	Respiratory manifestations	Gastrointestinal manifestations	MP IgM	Cold agglutinin	Acute pancreatitis diagnosis	Management
Yang et al. [3]	6/F	Cough, fever	Epigastric pain, tenderness, vomiting	Present	Present	Amylase, CT abdomen	Symptomatic, clarithromycin
Al-Abbasi et al. [4]	9/M	Cough, fever	Abdominal pain, vomiting	Present	Present	Amylase, CT abdomen	Symptomatic, piperacillin
Valdés Lacasa et al. [5]	21/M	Upper respiratory infection	Epigastric pain	Present	Present	Amylase, lipase	Symptomatic, amoxicillin, clavulanate
Nakagawa et al. [6]	8/F	Pneumonia	Abdominal pain	Present	NR	CT, amylase	Clarithromycin, gabexate mesylate
Benzaquen et al. [7]	28/F	Pneumonia	Abdominal pain	Present	Present	CT, amylase	Symptomatic, clarithromycin

The pathophysiology of pancreatitis injury due to *M. pneumoniae* infection is not understood yet; however, a few mechanisms have been proposed. *M. pneumoniae* can spread hematogenously to distant sites and induce the release of inflammatory cytokines at the local site of infection, which causes direct extrapulmonary manifestations [12]. Immunological reactions between *M. pneumoniae* and immune cells leading to activation of the immune system may also trigger the inflammatory response causing indirect pancreatic injury by expressing glycolipids and glycoproteins to the cell surface [6,7]. Another mechanism of extrapulmonary manifestation could be vasculitis or thrombosis with or without activation of the systemic coagulation cascade resulting in pancreatic injury [13].

Diagnosis of acute pancreatitis requires two out of three of the following: epigastric pain (radiating to the back), elevated lipase or amylase (more than three times the upper limit), and CT findings of pancreatic inflammation [10]. The patient was diagnosed with acute pancreatitis and interstitial pneumonia in our case. Modified Glasgow score was 2/8, consistent with mild to moderate acute pancreatitis. The patient had concurrent acute pancreatitis and lung infection, and we believe that pneumonia in our case is not likely to be considered a direct consequence of acute pancreatitis. Additionally, interstitial pneumonia and acute pancreatitis improved quickly after starting antibiotic therapy. We assume that the chronological association of interstitial pneumonia and pancreatitis, the presence of cold agglutinin, and the effective clinical response to appropriate antibiotics support the notion that *M. pneumoniae* is the cause of both diseases in the absence of other causative agents.

## Conclusions

Despite its rarity, *M. pneumoniae* can cause extrapulmonary manifestations, including acute pancreatitis. *M. pneumoniae* should be considered a differential diagnosis among the infectious etiology of acute pancreatitis when no other causative agent is identified. As *M. pneumoniae* presents with complex symptoms, aggressive supportive care and close observation are essential to prevent mortality and morbidity in acute pancreatitis. Our case highlights a possible link between *M. pneumoniae* and pancreatitis, and this evidence is supported by clinical, serological, and radiological features.
